# Efficiency analysis of nutritional screening tools for children with congenital heart disease: a retrospective observational study

**DOI:** 10.3389/fnut.2025.1572805

**Published:** 2025-06-30

**Authors:** Ying Xu, Yingying Jiang, Minzhi Guo, Yiping Wang, Hongmiao Huang, Jiaqian Xie, Dongshan Liao

**Affiliations:** ^1^Department of Clinical Nutrition, Fujian Medical University Union Hospital, Fuzhou, China; ^2^Department of Occupational Health, Fujian Center for Prevention and Control of Occupational Diseases and Chemical poisoning, Fuzhou, China; ^3^Department of Cardiovascular Surgery, Fujian Medical University Union Hospital, Fuzhou, China; ^4^Department of Food and Nutritional Sciences, University of Ottawa, Ottawa, ON, Canada; ^5^Fujian Provincial Center for Cardiovascular Medicine, Fuzhou, China

**Keywords:** congenital heart disease, nutritional screening, malnutrition, STAMP, STRONGkids

## Abstract

**Objective:**

This study aims to (1) determine the prevalence of malnutrition among hospitalized children with congenital heart disease (CHD), (2) evaluate the effectiveness of common pediatric nutritional screening tools across various age groups, and (3) specifically assess the tools’ efficacy in identifying severe malnutrition, thereby facilitating early nutritional intervention.

**Methods:**

A retrospective observational analysis was performed using clinical data from 3,677 children (0–18 years) with congenital heart disease who underwent surgical intervention at the Cardiothoracic Surgery Center between January 2018 and December 2022. The World Health Organization growth curves were used as standards to compare the efficacy of four screening tools: Screening Tool for the Assessment of Malnutrition in Pediatrics (STAMP), Screening Tool Risk on Nutritional status and Growth (STRONGkids), Risk Adjustment in Congenital Heart Surgery-1 method (RACHS-1), and the combined STAMP + STRONGkids (SS) adjusted score across different age groups. Categorical data were expressed as percentages, and Chi-square tests were used for statistical analysis, with pairwise comparisons performed using Bonferroni correction. Receiver Operating Characteristic (ROC) curves were employed to calculate specificity, sensitivity, and optimal cutoff values. The consistency of screening results was further assessed using Youden’s index and Kappa values.

**Results:**

The prevalence of malnutrition among CHD patients was 32.9% (1,208/3,667). Using World Health Organization (WHO) curves as the gold standard for diagnosing malnutrition, the AUC for the ROC curves of STAMP, STRONGkids, and SS were 0.841, 0.747, and 0.863 (*P* < 0.01), respectively, for nutritional risk screening among CHD patients. Optimal Youden indices were achieved at a STAMP score of 3.5 (55.9%), a STRONGkids score of 2.5 (41.5%), and an SS score of 3.25 (64.5%). Age-based subgroup analysis revealed that STAMP had the best sensitivity of 70.1% (Negative Prediction Rate (NPV) 96.1) at a score of 3.5 for children aged 6–18 years, STRONGkids showed optimal sensitivity of 78.1% (NPV 77.3) at a score of 2.5 for infants aged 0–1 year, and SS demonstrated 74.7% sensitivity (NPV 96.6) at a score of 3.25 for children aged 6–18 years. Further analysis indicated that STAMP at a score of 2.5 and SS at a score of 2.75 showed balanced sensitivity and specificity across all age groups. Additionally, for different degrees of nutritional deficiencies, STAMP at 3.5 and SS at 3.25 demonstrated ideal specificity, with all Kappa values being *P* < 0.001.

**Conclusion:**

For hospitalized CHD patients aged 0–18 years, the nutritional screening tool STAMP is more effective than STRONGkids, and SS combines the advantages of both tools as it demonstrates the best screening efficacy. However, the area under the ROC curve (AUC) for the RACHS-1 score was 0.525 (*P* >0.01), indicating not suitable for nutritional risk screening. To enhance sensitivity in screening malnutrition in CHD patients, the recommended cutoff values are 3 for STAMP and 2.5 for SS. For identifying severe malnutrition, STAMP at a cutoff of 3.5 and SS at 3.25 show higher overall screening efficacy.

## Introduction

Malnutrition in hospitalized children significantly affects both length of stay (LOS) and mortality rates (1, 2), placing a substantial economic burdens on families and strains national healthcare resources and socioeconomic systems (3, 4). Congenital heart disease (CHD), characterized by abnormal fetal development of the heart and major vessels, is the most prevalent congenital anomaly in China. CHD detection rates range between 2.9‰ and 16.0‰ across various regions. Currently, approximately two million individuals live with CHD in China, and around 150,000 new cases emerge annually, with 30–40% classified as complex CHD (5). Children with CHD are especially vulnerable to severe malnutrition due to chronic hypoxemia, exacerbating their clinical prognosis (6–8). Previous studies have demonstrated a significant negative correlation between malnutrition severity in CHD patients and adverse clinical outcomes, including increased LOS, elevated mortality rates, higher postoperative infection rates, prolonged intensive care unit (ICU) stays, extended mechanical ventilation duration, acute renal failure, and the necessity of postoperative inotropic medications (9–12). While standard anthropometric indicators such as height, weight, and Body Mass Index (BMI) Z-scores are commonly utilized globally, they inadequately detect early-stage malnutrition or those at risk due to acute conditions (13). Recognizing these limitations has prompted repeated recommendations to incorporate specialized nutritional screening tools into routine clinical practice for hospitalized children (14, 15).

Nutritional screening tools facilitate the timely identification of pediatric patients at risk of malnutrition, enabling early nutritional intervention. Prominent screening instruments include the Screening Tool for the Assessment of Malnutrition in Pediatrics (STAMP), Screening Tool Risk on Nutritional status and Growth (STRONGkids), Pediatric Yorkhill Malnutrition Score (PYMS), and Pediatric Nutritional Screening Score (PNSS). Each tool features unique methodological frameworks, target populations, and contexts of use (16–20). Although STAMP and STRONGkids offer foundational structures ideal for widespread use, PNSS and PYMS require more specialized settings with experienced screening personnel. Currently, no consensus exists regarding the optimal screening tool for hospitalized pediatric populations, underscoring the need for further evaluation (14, 21–23).

Selecting suitable nutritional screening tools for hospitalized CHD patients requires consideration of clinical environments, available resources, and patient-specific conditions. STAMP (for ages 2–16 years) and STRONGkids (for infants from 1 month to 18 years) are widely adopted due to their ease and rapid application (24, 25). However, STRONGkids includes subjective clinical assessments performed exclusively by experienced pediatricians, potentially causing workload imbalances and variability in results (16). Such limitations necessitate tailored approaches and careful adaptation for CHD-specific contexts. Additionally, the RACHS-1 (Risk Adjustment in Congenital Heart Surgery-1) score, traditionally used to estimate surgical risk, provides valuable insights into anticipated disease severity and recovery needs, crucial for planning comprehensive nutritional interventions (8, 26, 27).

Given these considerations, this study simultaneously applied STAMP, STRONGkids, RACHS-1, and a combined adjusted score (STAMP + STRONGkids, SS) to hospitalized CHD patients aged 0–18 years. Using WHO growth curves as benchmarks, we evaluated the prevalence of malnutrition and compared the predictive accuracy and sensitivity of these screening tools. Our goal was to establish effective screening practices for early detection and intervention.

We hypothesize that:

1. Single nutritional screening tools (STAMP, STRONGkids, or RACHS-1) demonstrate limited effectiveness when applied independently in hospitalized CHD patients.
2. The combined screening score (SS) offers superior predictive performance compared to individual screening tools.
3. Optimal nutritional risk cutoff values must be age-specific to enhance screening accuracy across different CHD age groups.
4. Screening cutoff values should also be adapted to clearly distinguish between the identification of general versus severe malnutrition.

## Materials and methods

### Study design and patients

This study analyzed clinical data from 3,677 children with congenital heart disease (CHD), aged 0–18 years, who underwent surgical treatment at the Cardiothoracic Surgery Center of Fujian Medical University Union Hospital between January 2018 and December 2022. Initially, 4,489 cases were identified via the hospital’s clinical data platform. After applying inclusion and exclusion criteria, 3,677 patients were included in the final analysis. Inclusion criteria: (1) Age between 0 and 18 years. (2) Confirmed diagnosis of CHD based on WHO diagnostic criteria, and having undergone surgical treatment (either open-heart or interventional procedures). (3) Compliance with institutional ethical standards and availability of informed consent from patients’ guardians. Exclusion criteria: (1) bnormal liver or kidney function or evidence of major organ dysfunction upon admission. (2) Diagnosed with genetic metabolic disorders or developmental syndromes affecting growth. (3) Presence of severe psychiatric illness or cognitive impairment. (4) Diagnosis of malignancy. The study was approved by the Ethics Committee of Fujian Medical University Union Hospital (Approval No. 2023KJT077).

### Assessment

Historical medical records were reviewed to extract STRONGkids and RACHS-1 scores. Based on these data, STAMP scores and the composite SS score—defined as the arithmetic mean of STAMP and STRONGkids scores—were calculated. The STRONGkids tool assesses four domains: (1) current nutritional status, (2) clinical symptoms or underlying diseases, (3) recent changes in nutritional intake and output, and (4) recent weight changes. The total score ranges from 0 to 5, with scores ≥ 4 indicating a high risk of malnutrition. The STAMP tool evaluates three domains: (1) underlying disease, (2) nutritional intake, and (3) anthropometric percentiles for weight and height. The total score ranges from 0 to 9, and scores ≥ 4 are considered indicative of high nutritional risk. RACHS-1 system classifies congenital cardiac surgical procedures into six categories based on complexity, with scores ranging from 0 to 6 (27). The SS score was defined as (STAMP + STRONGkids)/2. The screening scales are presented in Supplementary Appendices 1–3.

Malnutrition classification was based on WHO Z-score criteria using the WHO Anthro software. For children aged 0–5 years, height-for-age (HFA), weight-for-age (WFA), and weight-for-height (WFH) Z-scores were used; for those aged > 5 years, BMI-for-age Z-score (BMI Z-score) was calculated. Grading standards for malnutrition included: Weight-for-age (WFA) or BMI Z < −2 is defined as underweight, height-for-age (HFA) < −2 indicates stunting, weight-for-height (WFH) < −2 indicates wasting, WFA > 2 or BMI Z > 1 indicates overweight, and WFA > 3 or BMI Z > 2 indicates obesity. Children categorized as underweight, stunted, wasted, or overweight are considered malnourished, while Z < −3 or Z > 3 indicates severe malnutrition (13). Anthropometric measurements (height/length and weight) were obtained upon admission by trained pediatric nurses using calibrated, standardized equipment. All screening tools were completed and scored by clinically trained physicians and registered dietitians who had undergone unified training. To ensure accuracy and reproducibility, all assessments were independently reviewed by two professionals.

### Statistical analyses

Analyses were performed using SPSS 25.0 software. Categorical data were expressed as percentages, and Chi-square tests were used for statistical analysis, with pairwise comparisons conducted using the Bonferroni method. Receiver Operating Characteristic (ROC) curves were utilized to calculate specificity, sensitivity, negative predictive value (NPV), positive predictive value (PPV), and cutoff values. Youden’s index and Kappa values were employed to assess the consistency of screening results. A *P*-value < 0.05 was considered statistically significant.

## Results

A total of 3,667 patients were enrolled in this study, with a prevalence of malnutrition at 32.9% (1,208/3,667). Among male patients, the malnutrition rate was 32.4% (601/1,854), while among female patients, it was 33.5% (607/1,813), with no statistically significant difference observed between sexes (*P* > 0.05).

Patient distribution varied across age groups, with the number of cases gradually decreasing with age. The prevalence of malnutrition was highest in the 0–1 year age group (45.6%, 598/1,312), followed by the 3–6 years group (34.2%, 232/679), 1–3 years group (28.5%, 303/1,062), and the 6–18 years group (12.2%, 75/614). The differences among age groups were statistically significant (*P* < 0.01).

Regarding disease classification, 362 patients (9.9%) were diagnosed with cyanotic CHD. The prevalence of malnutrition in this subgroup was significantly higher at 45.9% (166/362), compared to 31.5% (1,042/3,305) in patients with acyanotic CHD (*P* < 0.01) (Table 1).

**TABLE 1:** Baseline characteristics of children with congenital heart disease.

Patient characteristics	Number (n)	Malnutrition [n (%)]	Normal Nutrition [n (%)]	χ ^2^ value	*P*-value
Total	3,667	1,208 (32.9)	2,459 (67.1)		
Gender				0.470	0.493
Male	1,854	601 (32.4)	1,253 (67.6)		
Female	1,813	607 (33.5)	1,206 (66.5)		
Age Group*				224.075	< 0.001
0–1 year	1,312	598 (45.6)	714 (54.4)		
1–3 years	1,062	303 (28.5)	759 (71.5)		
3–6 years	679	232 (34.2)	447 (65.8)		
6–18 years	614	75 (12.2)	539 (87.8)		
Type of CHD*				30.322	< 0.001
Cyanotic	362	166 (45.9)	196 (54.1)		
Acyanotic	3,305	1,042 (31.5)	2,263 (68.5)		

*Significant differences were found between age groups (*P* < 0.001).

**FIGURE 1:**
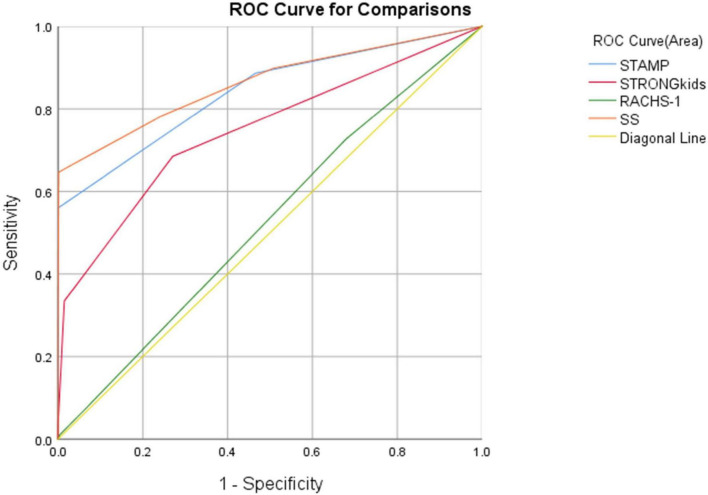
ROC curves for the four screening tools.

Using WHO standards as the gold standard for malnutrition diagnosis, ROC curves were plotted for four screening tools, revealing significant statistical differences among STAMP, STRONGkids, and SS in nutritional risk screening for CHD patients (*P* < 0.01). The AUC values and ROC curves are presented in Figure 1 and Table 2.

**TABLE 2:** Area under the curve (AUC) of different nutritional screening tools.

Screening Tools	AUC	Standard Error	*P*-Value	95%Confidence Interval (CI)
STAMP	0.841	0.008	<0.001	0.826∼0.856
STRONGkids	0.747	0.009	<0.001	0.729∼0.766
RACHS-1	0.525	0.010	0.013	0.505∼0.545
SS	0.863	0.007	<0.001	0.848∼0.877

We validated the four screening tools with different cutoff points, finding that STAMP demonstrated the best Youden index at a cutoff of 3.5 (55.9%). STRONGkids showed an optimal Youden index at a cutoff of 2.5 (41.5%). Notably, STAMP outperformed STRONGkids in both sensitivity and specificity. The RACHS-1 scoring did not perform well, with a maximum Youden index being merely 4.7% across different score thresholds. SS exhibited the best performance with cutoff values between 2.75 and 3.25, where it consistently showed good sensitivity and specificity. The SS score reached an optimal Youden index of 64.5% at a cutoff of 3.25. Detailed results can be found in Table 3.

**TABLE 3:** Validation of different cutoff values for screening scales.

Screening tools	Cut-off value	Sensitivity (%)	Specificity (%)	Youden index (%)
STAMP	2.5	88.7	53.4	42.1
	3.5	56.0	99.9	55.9
	4.5	43.8	99.9	43.7
	5.5	8.6	100.0	8.6
STRONGkids	2.5	68.5	73.0	41.5
	3.5	33.4	98.5	31.9
	4.5	3.7	100.0	3.7
RACHS-1	1.5	72.7	32.0	4.7
	2.5	6.4	94.4	0.8
	3.5	1.1	99.6	0.7
SS	2.25	89.8	49.3	39.1
	2.75	78.2	75.6	53.8
	3.25	64.7	99.8	64.5
	3.75	42.9	99.9	42.8
	4.25	16.1	100.0	16.1
	4.75	7.4	100.0	7.4
	5.25	3.6	100.0	3.6

Additionally, based on the optimal Youden index cutoff values, we analyzed the three well-performing screening tools across different age groups. Results indicated that STAMP at a cutoff of 3.5 had the highest sensitivity of 70.7% (NPV 96.1) for the 6–18 year group, STRONGkids at a cutoff of 2.5 showed the highest sensitivity of 78.1% (NPV 77.3) for the 0–1 year group, and SS at a cutoff of 3.25 demonstrated 74.7% sensitivity (NPV 99.6) for the 6–18 year group. Detailed findings are shown in Table 4.

**TABLE 4:** Comparison of three screening methods (based on optimal youden index) across different age groups.

Screening tools	Screening results	0–18 y	0–1 y	1–3 y	3–6 y	6–18 y
STAMP (3.5)	Sensitivity (%)	56.0	62.7	44.2	49.6	70.7
	Specificity (%)	99.9	100.0	100.0	99.6	100.0
	PPV	99.7	100.0	100.0	98.3	100.0
	NPV	82.2	76.2	81.8	79.2	96.1
	Kappa*	0.630	0.647	0.531	0.558	0.809
STRONG kids (2.5)	Sensitivity (%)	68.5	78.1	60.7	52.6	72.0
	Specificity (%)	73.0	62.3	75.60	80.50	77.0
	PPV	55.4	63.5	49.9	58.4	30.3
	NPV	82.5	77.3	82.8	76.6	95.2
	Kappa*	0.391	0.397	0.341	0.339	0.308
SS (3.25)	Sensitivity (%)	64.7	72.7	54.1	54.30	74.7
	Specificity (%)	99.8	99.9	100.0	99.30	100.0
	PPV	99.5	99.8	100.0	97.7	100.0
	NPV	85.2	81.4	84.5	80.7	96.6
	Kappa*	0.708	0.742	0.628	0.601	0.838

*All Kappa values were *P* < 0.001.

Based on the results from the optimal Youden index cutoff values, we selected STAMP and SS and adjusted the cutoff values to achieve higher sensitivity and NPV for screening results among different age groups of CHD patients. The data indicated that STAMP at a cutoff of 2.5 improved sensitivity across all age groups, with sensitivities and NPVs of 91.6 and 85.4%, 83.2 and 87.9%, 84.% and 87.8%, and 100.0 and 100.0%, respectively. SS at a cutoff of 2.75 exhibited balanced sensitivity and specificity across age groups, yielding 85.3 and 85.4%, 71.3 and 87.0%, 67.2 and 82.9%, and 84.0 and 97.3%, respectively. At a cutoff of 2.25, sensitivity increased significantly, though specificity declined, yielding results of 93.0 and 86.0%, 84.5 and 88.5%, 85.3 and 87.8%, and 100.0 and 100.0%, respectively. All Kappa values were < 0.001, detailed in Table 5.

**TABLE 5:** Comparison of two screening tools (post cutoff adjustment) across different age groups.

Screening tools	Screening results	0–18y	0–1y	1–3y	3–6y	6–18y
STAMP (2.5)	Sensitivity (%)	88.0	91.6	83.2	84.5	100.0
	Specificity (%)	53.4	41.0	48.7	57.7	72.9
	PPV	48.3	56.6	39.3	50.9	33.9
	NPV	90.6	85.4	87.9	87.8	100.0
	Kappa*	0.347	0.311	0.239	0.364	0.397
SS (2.75)	Sensitivity (%)	78.2	85.3	71.3	67.2	84.0
	Specificity (%)	75.6	66.9	76.7	82.6	80.0
	PPV	61.2	68.4	55	66.7	36.8
	NPV	87.6	85.4	87	82.9	97.3
	Kappa*	0.503	0.512	0.44	0.497	0.412
SS (2.25)	Sensitivity (%)	89.8	93.0	84.5	85.3	100.0
	Specificity (%)	49.3	36.3	47.6	54.6	64.6
	PPV	46.5	55.0	39.1	49.4	28.2
	NPV	90.8	86.0	88.5	87.8	100.0
	Kappa*	0.316	0.277	0.238	0.340	0.308

*All Kappa values were *P* < 0.001.

Grading malnutrition severity according to WHO standards aimed to compare the efficacy of the two screening tools in identifying severe malnutrition, focusing on specificity. The comparison at different score levels across age groups indicated that STAMP at a cutoff of 3.5 had higher specificity, with the following specificity and PPV for each group: 83.9 and 56.3%, 93.6 and 54.5%, 91.7 and 59.0%, and 94.3 and 35.8%. For SS, as the cutoff decreased from a of 3.25 to 2.25, with gradually increasing sensitivity (except in the 6–18 year group), yielding specificity and PPV of 80.9 and 55.3%, 90.9 and 47.0%, 90.3 and 56.6%, and 93.8 and 33.9%. All Kappa values were *P* < 0.001, detailed in Table 6.

**TABLE 6:** Comparison of two screening tools for malnutrition severity (based on different scores) across age groups.

Screening tools	Screening results	0–18 y	0–1 y	1–3 y	3–6 y	6–18 y
STAMP (3.5)	Sensitivity (%)	72.2	72.3	69.5	69.7	100.0
	Specificity (%)	90.3	83.9	93.6	91.7	94.3
	PPV	54.8	56.3	54.5	59.0	35.8
	NPV	95.2	91.4	96.6	94.7	100.0
	Kappa*	0.551	0.510	0.562	0.571	0.505
STAMP (2.5)	Sensitivity (%)	95.3	95.9	93.3	94.9	100.0
	Specificity (%)	45.3	32.5	43.3	49.8	66.1
	PPV	22.5	28.9	15.3	24.4	8.6
	NPV	98.3	96.5	98.3	98.3	100.0
	Kappa*	0.171	0.155	0.112	0.204	0.107
SS (3.25)	Sensitivity (%)	79.6	82.5	73.3	73.7	100.0
	Specificity (%)	88.1	80.9	90.9	90.3	93.8
	PPV	52.2	55.3	47.0	56.6	33.9
	NPV	96.4	94.2	96.9	95.3	100.0
	Kappa*	0.555	0.539	0.514	0.569	0.483
SS (2.75)	Sensitivity (%)	90.7	93.2	89.5	82.8	100.0
	Specificity (%)	65.8	53.5	68.8	73.8	74.5
	PPV	30.2	36.5	23.9	35.0	11.1
	NPV	97.7	96.5	98.4	96.2	100.0
	Kappa*	0.308	0.300	0.262	0.362	0.153
SS (2.25)	Sensitivity (%)	96.7	97.6	95.2	94.9	100.0
	Specificity (%)	41.8	28.8	42.1	47.1	58.8
	PPV	21.4	28.2	15.3	23.4	7.1
	NPV	98.7	97.7	98.8	98.2	100.0
	Kappa*	0.156	0.141	0.112	0.186	0.080

*All Kappa values were *P* < 0.001.

## Discussion

### Impact and importance of malnutrition

Malnutrition is a major contributor to morbidity and mortality in hospitalized children, leading to prolonged hospital stays, increased healthcare costs, and poorer clinical outcomes (3, 4). Its prevalence can reach up to 51% in pediatric inpatients in developed and transitional countries (15). In children with congenital heart disease (CHD), malnutrition is particularly concerning due to elevated metabolic demands and frequent feeding difficulties (8). It has been linked to adverse postoperative outcomes, including increased infection rates, longer ICU stays, extended mechanical ventilation, higher inotropic drug use, and mortality (9, 11). Additionally, malnutrition may impair long-term motor and neurodevelopmental outcomes (28). In our study, 32.9% of CHD patients were malnourished, with the highest rate (45.6%) in infants aged 0–1 year. These findings highlight the urgent need for early nutritional assessment and intervention to optimize recovery and reduce complications.

### Selection and evaluation of screening tools

Effective malnutrition screening in hospitalized children requires tools with both high sensitivity and specificity. While STAMP, STRONGkids, and RACHS-1 are commonly used, their performance varies across clinical contexts. In this CHD-specific study, we evaluated these tools alongside the combined score SS, which integrates STAMP and STRONGkids by averaging their outputs.

Although RACHS-1 is useful for surgical risk stratification (27), its value in nutritional screening was limited, with an AUC of 0.525 (95% CI: 0.505–0.545, *P* = 0.013). In contrast, SS demonstrated superior discriminatory ability with the highest AUC of 0.863, outperforming both STAMP (0.841) and STRONGkids (0.747), particularly at a cutoff of 3.25. These results support the use of SS as a more effective and clinically applicable tool for nutritional risk screening in pediatric CHD populations.

### Age-specific screening performance

Our study included CHD patients aged 0–18 years and revealed significant age-related differences in malnutrition prevalence, with the highest rates observed in infants (0–1 years) and preschool children (3–6 years). STAMP demonstrated optimal sensitivity in the 6–18 years group (70.7%, NPV 96.1), whereas STRONGkids performed best in the 0–1 years group (78.1%, NPV 77.3). The combined score SS showed the highest sensitivity in the 6–18 years group (74.7%, NPV 99.6). These findings underscore the importance of age-specific calibration of screening tools to improve diagnostic accuracy and better target nutritional interventions in CHD populations.

### Evaluation of the adjusted screening tools

Adjustment of cutoff values notably improved the clinical utility of STAMP and SS across different age groups. For instance, STAMP at a cutoff of 2.5 achieved a high sensitivity of 88.0% and an NPV of 90.6%, though at the expense of reduced specificity—highlighting the inherent trade-off between sensitivity and specificity. SS at a cutoff of 2.75 offered a better balance, making it a more reliable tool for early intervention.

In identifying severe malnutrition, the adjusted tools demonstrated enhanced diagnostic performance. SS at a cutoff of 3.25 showed excellent sensitivity and specificity across age groups, reinforcing its value in detecting high-risk cases.

These findings suggest that appropriate threshold adjustments significantly enhance the practicality and accuracy of screening tools. However, potential overestimation of nutritional risk warrants further validation in broader and more diverse clinical populations. Future research should explore the contextual adaptation of these tools to optimize their performance across varying pediatric cohorts.

### Limitations of the study

While the approach of averaging scores to create the SS (STAMP + STRONGkids) composite score notably improved its screening performance, several limitations should be acknowledged. First, the study’s single-center, retrospective design may introduce selection bias; future multicenter, prospective studies are needed to validate these findings. Second, each screening tool assesses distinct dimensions of malnutrition risk: STAMP evaluates dietary intake, growth status, and disease risk, whereas STRONGkids includes subjective clinical assessment, disease severity, nutritional intake and loss, and recent weight changes. In the present study, we did not analyze or score each individual item within these tools separately. Future research should aim to refine the composite model by employing more sophisticated approaches–such as decision tree algorithms or machine learning models–to determine the relative importance of specific assessment domains. Adjusting the weighting coefficients accordingly may enhance both the efficiency and usability of the SS tool in clinical settings. Finally, emerging screening tools developed by other research groups (29) offer alternative frameworks and should be included in future comparative validation studies to identify the most effective and practical tools for pediatric CHD populations.

### Clinical practice recommendations

In clinical implementation, we recommend the routine use of the adjusted screening tools, such as SS, in both preoperative and postoperative phases. Preoperative screening should focus on early identification of high-nutritional-risk patients to allow timely nutritional interventions. Postoperative care should involve regular assessments to adjust nutritional support as needed. Individualized nutritional interventions for CHD patients should be tailored according to their specific needs and circumstances, which is crucial to improving outcomes. Implementation of these strategies in clinical practice of comprehensive nutritional management can facilitate patient recovery, reduce the incidence of complications, and support overall growth and development. This provides a theoretical basis for developing personalized nutritional intervention strategies.

## Conclusion

For hospitalized CHD patients aged 0–18 years, the nutritional screening tool STAMP demonstrates superior screening efficacy compared to STRONGkids. SS, which integrates the strengths of both screening tools, shows promising screening performance. The RACHS-1 scoring system is not suitable for nutritional risk screening. To enhance sensitivity in malnutrition screening for CHD patients, we recommend a cutoff value of 2.5 for STAMP and 2.25 for SS. Additionally, for identifying severe malnutrition, STAMP at a cutoff of 3.5 and SS at a cutoff of 3.25 provide higher overall screening efficacy.

These findings support the clinical utility of age-adjusted, purpose-specific screening thresholds and highlight the value of combined screening approaches for early nutritional risk detection and intervention in CHD populations.

## Data Availability

The datasets for this article are not publicly available due to concerns regarding participant/patient anonymity. Requests to access the datasets should be directed to the corresponding author.
