# Supplementary material for: Efficiency analysis of nutritional screening tools for children with congenital heart disease: a retrospective observational study
Source: Front Nutr. 2025 Jun 30;12:1572805. doi: 10.3389/fnut.2025.1572805 (PMC12256225; doi:10.3389/fnut.2025.1572805)
Supplement: Supplementary file 1 [file Image_1.pdf]

OUTCOMES

Risk Adjustment for Congenital Heart Surgery: The RACHS-1 Method

Kathy J. Jenkins

The new health care environment has increased the need for accurate information about outcomes after pediatric cardiac surgery to facilitate quality improvement efforts both locally and globally. The Risk Adjustment for Congenital Heart Surgery (RACHS-1) method was created to allow a refined understanding of differences in mortality among patients undergoing congenital heart surgery, as would typically be encountered within a pediatric population. RACHS-1 can be used to evaluate differences in mortality among groups of patients within a single dataset, such as variability among institutions. It can also be used to evaluate the performance of a single institution in comparison to other benchmark data, provided that complete model parameters are known. Underlying assumptions about RACHS-1 risk categories, inclusion and exclusion criteria, and appropriate and inappropriate uses are discussed.
© 2004 Elsevier Inc. All rights reserved.

Key words: Pediatric cardiac surgery outcomes, congenital heart surgery, risk-adjusted mortality.

Health outcomes have been increasingly scrutinized as the best source of information about how to deliver optimal medical treatment. Indeed, evaluation of health care practice without outcome assessment can be readily criticized as lacking relevance. However, outcome evaluation, even for easily measured outcomes such as in-hospital mortality, requires data collection and complex analytical techniques for interpretation. Although some analytical tools have been developed to provide system-wide information about medical care delivery, refined evaluation of outcomes for particular conditions requires specifically designed analytical techniques to account for the important nuances inherent in delivery of certain types of services.

Pediatric cardiac surgery is practiced increasingly as a part of a national, or even international, health care delivery system. Features of modern society, such as ready access to information through the internet, ease of travel, etc, have allowed parents and responsible physicians to seek care from a broad range of providers, far beyond more traditional geographic boundaries. Practitioners have long sought to improve outcomes for congenital heart disease through individualized efforts and academic investigation. The new health care environment has increased the need for accurate information about outcomes after pediatric cardiac surgery to facilitate quality improvement efforts both locally and globally.

The Risk Adjustment for Congenital Heart Surgery (RACHS-1) method was created to allow a refined understanding of differences in mortality among patients undergoing congenital heart surgery, as would typically be encountered within a pediatric population. The details of the development and initial validation have been published previously.[1] To summarize, RACHS-1 was created using a judgment-based approach using input from a nationally representative panel of expert pediatric cardiologists and surgeons, who sought to develop a method that would provide accurate comparisons of outcomes for groups of patients undergoing congenital heart surgery using information that did not require excessive primary data collection. The panel chose to focus on short-term mortality after surgery, because of its importance, and selected in-hospital mortality

From the Department of Cardiology, Children's Hospital, Boston, MA.
Supported in part by grant no. National Heart, Lung, and Blood Institute K08-HL02936.
Address reprint requests to Kathy J. Jenkins, MD, MPH, Department of Cardiology, 300 Longwood Ave, Boston, MA 02115.
© 2004 Elsevier Inc. All rights reserved.
1092-9126/04/0701-0024$30.00/0
doi:10.1053/j.pcsu.2004.02.009

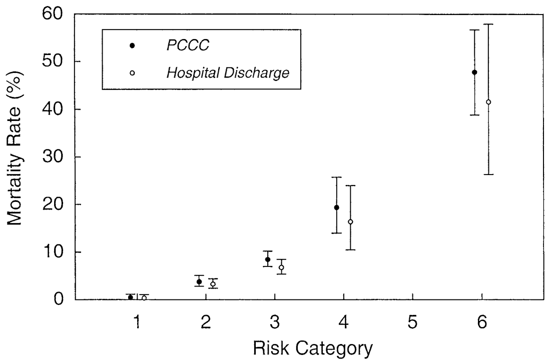

Figure 1. This figure shows observed mortality rates according to risk category, with 95% confidence limits, using data from the Pediatric Cardiac Care Consortium (closed circles) and statewide administrative data (open circles).[1]

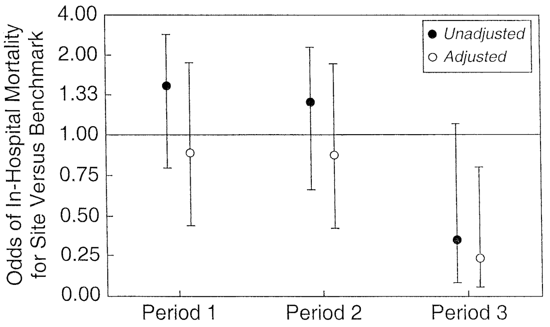

Figure 2. This figure shows unadjusted (closed circles) and adjusted (open circles) mortality data for a single center in Kansas, in comparison to contemporaneous population data for three time periods. The reduction in risk-adjusted mortality in time period 3 was after a change in referral pattern.[3]

because of the ease of availability of this endpoint in administrative and other datasets.

To apply RACHS-1, cardiac procedures are grouped into six risk categories, several of which are stratified by age or diagnosis. (Fig 1).[1] These risk categories are included in multivariable models, along with age (<30 days, 30 days to 1 year, and >1 year), prematurity, and the presence of a major noncardiac structural anomaly, such as cleft lip/palate or anal atresia (Table 1). Cardiac procedures during which more than one surgical procedure are performed simultaneously are placed in the risk category of the highest risk procedure, and a corrective factor is included in multivariable models.

Table 1. RACHS-1 Final Multivariable Model Using Data from Pediatric Cardiac Care Consortium

	Odds Ratio	*P* Value
Risk category 1	1.0	—
Risk category 2	6.6	<.001
Risk category 3	15.5	<.001
Risk category 4	28.2	<.001
Risk category 5	97.8	<.001
Risk category 6	93.4	<.001
Age ≤30 days	3.0	<.001
Age 31 days to 1 year	1.9	<.001
Age >1 year	1.0	—
Major structural anomaly	1.8	.011
Prematurity	1.8	.001
Combination procedures	1.5	.009

Applying RACHS-1 to Specific Research Questions

RACHS-1 can be used to evaluate differences in mortality among groups of patients within a single dataset, such as variability among institutions. Adjusted mortality rates can be calculated, by correcting observed mortality rates for more or less complex case mixes, in comparison to the average within the dataset. Alternatively, standardized mortality ratios can be calculated as the ratio of the observed deaths over the expected deaths, based on the average performance with similar cases within the dataset.[2] RACHS-1 can also be used to evaluate the performance of a single institution in comparison to other benchmark data, provided that complete model parameters are known (Fig 2).[3]

RACHS-1 can further be used to evaluate the independent effect of patient-level parameters, such as gender, race,[4] or insurance type[5] on in-hospital mortality. For these analyses, standard multivariable modeling techniques are used, with inclusion of the RACHS-1 categories and other variables in logistic regression models, along with the parameter of interest. Inclusion of RACHS-1 variables in multivariable analyses reduces confounding by case mix when making inferences about the primary analytical question. Similar models can be developed to evaluate institution-level variables, such as annual case volume or teaching status. However, in these instances,

more complex modeling techniques, such as generalized estimating equations, must be used to avoid attributing the effect of a specific institution on differences in mortality to the institutional attribute under study. These modeling techniques are more conservative than traditional models if there is a higher level of correlation of outcomes within institutions than between institution "classes," but approach standard modeling techniques if intra-institutional correlation is not present.[6]

Clarification of RACHS-1 Risk Categories

A major component of RACHS-1 is the grouping of individual types of cardiac procedures with similar risks for in-hospital death together into six risk categories. The risk categories were created by consensus of the panel using collective judgment and actual mortality data from two large datasets. Several important points regarding this component of RACHS-1 should be emphasized. First, a major goal in the development of RACHS-1 was the creation of a tool that would be useful in the assessment of outcomes across an entire hospital pediatric cardiac surgical caseload, rather than for a single cardiac procedure. Methods for single procedures may be more accurate to understand outcomes for that specific procedure, but do not provide insight into overall performance. To accomplish this, RACHS-1 needed to incorporate information from infants and children undergoing multiple types of surgery. As a general principle, risk adjustment methods should only include information that places patients at risk for certain health outcomes, not discretionary aspects of treatment, because these aspects of care are not "inherent to the patient." Thus, in most risk adjustment systems, patient diagnosis, not type of surgery, would be included. However, procedure type can be used as a surrogate for diagnosis, provided that a specific treatment course is universally applied when a specific diagnosis is encountered. This is the case for the vast majority of congenital heart defects undergoing surgery. For example, tetralogy of Fallot is repaired by "total repair of tetralogy of Fallot," meaning ventricular septal defect closure and right ventricular outflow tract reconstruction, with or without atrial septal defect closure or patent ductus arteriosus ligation. Using such broad definitions that do not include detailed information about surgical technique, there are very few "discretionary" approaches to most types of defects.

There were several nuances about congenital heart disease that made incorporating cardiac procedure rather than diagnosis preferable. Diversity of anatomy is the hallmark of congenital heart defects, and often exceeds the diagnostic information in most coding systems. However, in many instances, especially for patients with complex defects, diverse anatomic diagnoses are often treated with standard operative approaches, such as "Fontan-type operation" for complex single ventricle physiology or "placement of a right ventricular to pulmonary artery conduit" for complex right ventricular outflow tract obstruction. In terms of risk for in-hospital death, these patients can be grouped together based on the operative approach, despite their diverse anatomy. In addition, procedures may be more accurately coded in administrative databases because these codes are the basis for billing, although this remains untested. To preserve the validity of using procedure as a surrogate for anatomy when using RACHS-1, when different operative approaches to the same defect are encountered, such as a right ventricular to pulmonary artery conduit adaptation of the Norwood procedure for hypoplastic left heart syndrome, it is essential that both operative approaches be placed in the same RACHS-1 category.

The RACHS-1 risk categories are essentially a marked data reduction, taking the diversity of anatomy inherent in a pediatric cardiac surgical caseload, and reducing these anatomic differences to a six-item ordinal scale. This data reduction occurs in two steps: first, using cardiac surgical procedure as a surrogate for diagnosis, and second, grouping procedures together with a similar risk for mortality. This data reduction is a powerful feature of RACHS-1, especially in the pediatric cardiac surgery field, with inescapable limitations in sample sizes and extremes of anatomic diversity which are the hallmark of congenital heart disease. Grouping procedures together allows achievement of sample sizes which allow meaningful statistical comparisons, while accomplishing case mix adjustment. However, the RACHS-1 groupings cannot be regarded as perfectly accurate risk adjustors in all circum-

stances. Individual analyses may include residual case mix confounding, such as a higher prevalence of hypoplastic pulmonary arteries among tetralogy of Fallot patients in some patient groups, which may be influential in some circumstances. When differences in mortality are suggested by analyses using RACHS-1, it is important that the possibility of residual case mix confounding be considered as a possible explanation.

Clarification of the analytical purpose of the risk groups further explains two frequently misunderstood aspects of the categorization methodology. First, it is clear why the panel chose to retain risk category 5, even though this category has fewer procedures than the other categories. In the opinion of the panel, these few procedures were distinct in terms risk of in-hospital death from procedures in categories 4 or 6. Specifically, patients in category 5 were at a higher risk of dying than those in 4, and a lower risk than those in 6. Although it may be difficult to make inferential statements about category 5 patients in some circumstances because of small numbers, these patients will be appropriately adjusted for when making overall case mix comparisons. Overall comparisons would have reduced validity if category 5 patients were placed in either adjacent category.

It is also clear why the panel chose not to categorize some procedures. The initial procedure groupings were made, in part, using several common coding systems, including ICD-9-CM and CPT-4. Although a few rare procedures were inadvertently overlooked, in most cases the panel discussed and chose not to classify procedures with "vague" codes that were not sufficiently descriptive, such as "revision of procedure," or where a single code could be used for procedures with widely varying risks of death. The ICD-9-CM coding system, in particular, includes codes with little information about the actual surgery performed. In these instances, the code was judged by the panel to be sufficiently imprecise so as to preclude risk assessment, and these procedures were excluded. Once again, these decisions were made to preserve the validity of comparisons made using more informative coded procedures, which were nearly always categorized by the panel. Because most RACHS-1 analyses of ICD-9-CM coded data incorporate about 80% to 90% of the total pediatric cardiac surgical caseload, measures of performance using RACHS-1 risk adjustment can be regarded as reasonable surrogates for overall performance. However, residual confounding by case mix caused by differences in use of excluded codes among patient groups should be considered as a possible explanation for mortality differences identified using RACHS-1.

Clarification of Other RACHS-1 Components

Similar reasoning was used for other case mix exclusions incorporated into the RACHS-1 methodology. Because the purpose of RACHS-1 was to allow comparisons of mortality caused by congenital heart surgical procedures, cases with significant risk factors for death from noncardiac surgical causes, such as premature infants who underwent patient ductus arteriosus ligation, were not included. Similarly, situations where in-hospital mortality differences were less relevant as a basis for comparison, such as cardiac transplantation, were excluded. RACHS-1 comparisons are also made for children less than 18 years of age only because many older patients undergo repeat operations with widely varying risks for death, which can not be easily incorporated into risk groups. All of these exclusions are necessary to allow meaningful comparisons using RACHS-1 within the included caseload, and to thus preserve the validity of each analysis. These exclusions should not be interpreted to suggest that these types of cases are not relevant parts of an institutional caseload or experience, but only that the baseline risk for death for these cases cannot be easily adjusted for in multivariate models.

Limitations in Interpretation of Analyses Using RACHS-1

To appropriately use RACHS-1, it is important to understand that the method was not developed to do true "predictive modeling" for individual cases, but rather to allow meaningful comparisons of mortality for groups of patients undergoing congenital heart surgery. Predictive modeling attempts to assess the actual likelihood that individual patients will die, and might be expected to include numerous distinguishing features of individual cases that alter risk for death. Condi-

tions that occur rarely, or those that are likely to be evenly distributed within groups of patients of reasonable size, do not necessarily need to be incorporated into risk adjustment methods not intended to accurately predict mortality, such as RACHS-1. For example, although pulmonary artery diameter might be an important determinant of death after tetralogy of Fallot repair, this variable need not be included in RACHS-1, because similarly sized groups of children undergoing tetralogy of Fallot repair should have fairly similar "distributions" of pulmonary artery diameters. A similar case can be made regarding coronary artery anomalies for transposition of the great arteries. Obviously, when comparison groups are small, this assumption may not be met, allowing for possible residual confounding by these types of factors. As was stated previously, residual confounding by case mix should always be considered as a possible explanation for mortality differences found using RACHS-1. Although the RACHS-1 method was not developed as a predictive model, it did show reasonable calibration, as measured using a Hosmer-Lemeshow statistic, preformed as part of the initial validation.[1]

RACHS-2

The RACHS-1 method is an excellent research tool with acceptable measurement properties. Because the tool merely requires that certain variables be incorporated into analyses in certain ways, without specifying model parameters, it can be flexibly used to allow risk adjustment in more recent data sets, even though the initial validation was completed using data from 1993 to 1995. Even if risk groups converge as mortality rates decrease, use of the RACHS-1 method will retain validity because risk groups are entered into models as discrete, rather than ordinal, variables. Similarly, if one of patient characteristics currently part of RACHS-1, such as presence of a major noncardiac anomaly, is no longer an important determinant of mortality, there will be no loss of validity incurred by continuing to include this variable in multivariable models.

Despite these robust features of the RACHS-1 method, some details will eventually need to be revised to retain validity. For example, changes in relative mortality for specific procedures or the development of new procedures will require revisions in the current method. The designation of "1" within the RACHS-1 name was intended to suggest that additional methods would need to be created in the future, eg, RACHS-2, -3, etc. Fortunately, the development process used initially should make revisions relatively easy to accomplish, by reconvening the original or a similar panel of experts to invoke a similar judgment-based process using more recent information and assumptions.

References

1. Jenkins KJ, Gauvreau K, Newburger JW, et al: Consensus-based method for risk adjustment for congenital heart surgery. J Thorac Cardiovasc Surg 123:110-118, 2002
2. Jenkins KJ, Gauvreau K: Center-specific differences in mortality: preliminary analysis using the risk adjustment in congenital heart surgery (RACHS-1) method. J Thorac Cardiovasc Surg 124:97-104, 2002
3. Allen SW, Gauvreau K, Bloom B, et al: Evidence based referral results in significantly reduced mortality after congenital heart surgery. Pediatrics 112:24-28, 2003
4. Gonzalez PC, Gauvreau K, DeMone JA, et al: Regional racial and ethnic difference in mortality for congenital heart surgery in children may reflect unequal access to care. Pediatr Cardiol 24:103-108, 2003
5. DeMone JA, Gauvreau K, Piercey GE, et al: Effect of insurance on mortality risk for congenital heart surgery. Pediatr Cardiol 24:97-102, 2003
6. Liang KY, Zeger SL: Longitudinal data analysis using generalized linear models. Biometrika 73:13-22, 1986
