# Supplementary material for: Efficiency analysis of nutritional screening tools for children with congenital heart disease: a retrospective observational study
Source: Front Nutr. 2025 Jun 30;12:1572805. doi: 10.3389/fnut.2025.1572805 (PMC12256225; doi:10.3389/fnut.2025.1572805)
Supplement: Supplementary file 2 [file Image_2.pdf]

STAMP SCREENING FORM

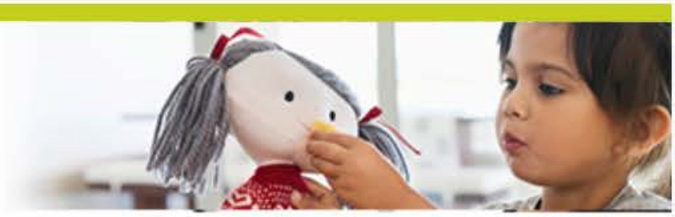

This form should be used with the centile quick reference tables or growth charts, and the weighing and measuring instructions. If you have any queries, please discuss them with a Dietitian.

STEP 1 - DIAGNOSIS

	Does the child have a diagnosis that has any nutritional implications? View diagnosis table	Score
☐	Definite nutritional implications	3
☐	Possible nutritional implications	2
☐	No nutritional implications	0

↓

STEP 2 - NUTRITIONAL INTAKE

	What is the child's nutritional intake?	Score
☐	No nutritional intake	3
☐	Recently decreased or poor nutritional intake	2
☐	No change in eating patterns and good nutritional intake	0

↓

STEP 3 - WEIGHT AND HEIGHT

	Use the centile quick reference tables to determine the child's measurements	Score
☐	> 3 centile spaces/≥3 columns apart (or weight < 2nd centile)	3
☐	> 2 centile spaces/= 2 columns apart	1
☐	0 to 1 centile spaces/columns apart	0

STAMP should be used in association with Trust referral guidelines and policies.
