# Supplementary material for: Efficiency analysis of nutritional screening tools for children with congenital heart disease: a retrospective observational study
Source: Front Nutr. 2025 Jun 30;12:1572805. doi: 10.3389/fnut.2025.1572805 (PMC12256225; doi:10.3389/fnut.2025.1572805)
Supplement: Supplementary file 3 [file Image_3.pdf]

STRONG$_{kids}$: Nutritional risk screening tool for children aged month – 18 years on admission to the hospital.

Screening risk of malnutrition<br>Asses following items < 24h after admission and once a week thereafter	Score →points	
1. Is there an underlying illness with risk for malnutrition (*see list*) or expected major surgery?	No	Yes → 2
2. Is the patient in a poor nutritional status judged with subjective clinical assessment: loss of subcutaneous fat and/or loss of muscle mass and/or hollow face?	No	Yes → 1
3. Is one of the following items present?<br>▪ Excessive diarrhoea (≥5 per day) and/ or vomiting (> 3 times/ day) during the last 1-3 days<br>▪ Reduced food intake during the last 1-3 days<br>▪ Pre-existing nutritional intervention (e.g. ONS or tube feeding)<br>▪ Inability to consume adequate nutritional intake because of pain	No	Yes → 1
4. Is there weight loss (all ages) and/or no increase in weight/height (infants < 1year) during the last few week-months?	No	Yes → 1

Maximum total score: 5 points

Diseases with risk of malnutrition (item 1)	
▪ Psychiatric eating disorder<br>▪ Burns<br>▪ Bronchopulmonary dysplasia (up to age 2 years)<br>▪ Celiac disease (active)<br>▪ Cystic fibrosis<br>▪ Dysmaturity/prematurity (until corrected age 6 months)<br>▪ Cardiac disease, chronic<br>▪ Infectious disease<br>▪ Inflammatory bowel disease<br>▪ Cancer	▪ Liver disease, chronic<br>▪ Kidney disease, chronic<br>▪ Pancreatitis<br>▪ Short bowel syndrome<br>▪ Muscle disease<br>▪ Metabolic disease<br>▪ Trauma<br>▪ Mental handicap/retardation<br>▪ Expected major surgery<br>▪ Not specified (classified by doctor)

Risk of malnutrition and need for intervention		
**Score**	**Risk**	**Intervention and follow-up**
4-5 points	**High risk**	• Consult doctor and dietician for full diagnosis and individual nutritional advice and follow-up.<br>• Check weight twice a week and evaluate nutritional advice<br>• Evaluate the nutritional risk weekly
1-3 points	**Medium risk**	• Consider nutritional intervention<br>• Check weight twice a week<br>• Evaluate the nutritional risk weekly
0 points	**Low risk**	• No nutritional intervention necessary<br>• Check weight regularly (according to hospital policy)<br>• Evaluate the nutritional risk weekly

Reference:
Hulst JM, Zwart H, Hop WC, Joosten KF. Dutch national survey to test the STRONGkids nutritional risk screening tool in hospitalized children. *Clin Nutr.* 2010;29(1532-1983; 0261-5614; 1):106-111.
